# Ethnic Variation in the Cross-sectional Association between Domains of Depressive Symptoms and Clinical Depression

**DOI:** 10.3389/fpsyt.2016.00053

**Published:** 2016-04-18

**Authors:** Shervin Assari, Ehsan Moazen-Zadeh

**Affiliations:** ^1^Department of Psychiatry, University of Michigan, Ann Arbor, MI, USA; ^2^Center for Research on Ethnicity, Culture, and Health, School of Public Health, University of Michigan, Ann Arbor, MI, USA; ^3^Medicine and Health Promotion Institute, Tehran, Iran; ^4^Mental Health Research Center, Tehran Psychiatric Institute, School of Behavioral Sciences and Mental Health, Iran University of Medical Sciences, Tehran, Iran

**Keywords:** ethnic groups, non-Hispanic Whites, African-Americans, depressive symptoms, depression, validity

## Abstract

**Background:**

The degree by which depressive symptoms and clinical depression reflect each other may vary across populations. The present study compared Blacks and Whites for the magnitude of the cross-sectional associations between various domains of depressive symptoms and endorsement of clinical disorders of depression.

**Methods:**

Data came from the National Survey of American Life, 2001–2003. We included 3570 Black (African-Americans) and 891 Non-Hispanic Whites. Predictors were positive affect, negative affect, and interpersonal problems measured using the 12-item Center for Epidemiologic Studies Depression Scale (CES-D). Outcomes were lifetime major depressive disorder (MDD), lifetime major depressive episode (MDE), 12-month MDE, 30-day MDE, and 30-day major depressive disorder with hierarchy (MDDH) based on the Composite International Diagnostic Interview (CIDI). Logistic regression models were applied in the pooled sample as well as Blacks and Whites.

**Results:**

Regarding CES-D, Blacks had lower total scores, positive affect, negative affect, and interpersonal problems compared to Whites (*p* < 0.05 for all comparisons). Blacks also had lower odds of meeting criteria for lifetime MDD and MDE, 12-month MDE, and 30-day MDE and MDDH (*p* < 0.05 for all comparisons). For most depressive diagnoses, ethnicity showed a positive and significant interaction with the negative affect and interpersonal problems domains, suggesting stronger associations for Blacks compared to Whites. The CES-D total score and CES-D positive affect domain did not interact with ethnicity on CIDI-based depressive diagnoses.

**Conclusion:**

Stronger associations between multiple domains of depressive symptoms and clinical depression may be due to higher severity of depression among Blacks, when they endorse the CIDI criteria for the disorder. This finding may explain some of previously observed ethnic differences in social, psychological, and medical correlates of depressive symptoms and clinical depression in the general population as well as clinical settings.

## Introduction

Depression is the fourth largest contributor to years lost due to disability (YLD) globally (1). Used for the measurement of prevalence of clinical depression, the World Health Organization Composite International Diagnostic Interview (CIDI) is a survey tool for population studies. As a combination of the Diagnostic Interview Schedule and the Present State Examination, the CIDI was developed for application by lay interviewers in measuring prevalence of recent or lifetime mental disorders based on DSM-IV-TR criteria (2–7). The CIDI has shown high concordance with the Structured Clinical Interview for DSM-IV (SCID) used by psychiatrists for diagnosing major depressive disorder (MDD) considering both receiver operating characteristic curve (AUC) analysis (2–4) and optimal diagnostic thresholds (5). The CIDI has shown consistent sensitivity and specificity among Latinos, non-Latino Blacks, and non-Latino Whites for diagnosis of MDD (8). Furthermore, it has shown validity for studying Blacks with different ethnicities (6, 7).

Besides MDD, scales that measure depressive symptoms provide useful information as they capture the burden of clinical and subclinical depression and also predict subsequent risk of MDD in the absence of the clinical disorder (1). As one of the most widely used self-reported measures of depressive symptoms in epidemiological studies, the Center for Epidemiologic Studies Depression Scale (CES-D) assesses different domains of depressive symptomatology, including depressed mood, feelings of guilt and worthlessness, and feelings of helplessness and hopelessness (9). The CES-D has shown high reliability, validity, and factorial invariance in population studies, including different ethnicities/races such as Blacks and Whites (9–20).

The degree by which depressive symptoms, measured using self-reported depressive methods, and endorsement of clinical MDD based on the CIDI overlap in the general population is still a matter of debate based on the importance of items and domains assessed by self-reported depressive symptoms scales (21). In addition to this concordance issue in studying the general population, ethnic differences have been reported concerning the association of self-rated mental health (SRMH) and CIDI-based diagnosis of psychiatric disorders (22, 23). Specifically, Blacks have shown a weaker association between a 5-point scaled question that asks “How would you rate your overall mental health?” and subsequent diagnosis of MDD compared to Whites. Furthermore, in recent longitudinal research on a nationally representative sample of Americans, the CES-D scale scores were predictive of endorsement of CIDI-based MDD criteria 15 years later only in Whites but not in Blacks (24). Therefore, despite the fact that both the CES-D (13–15) and the CIDI (25–27) are widely used methods for assessing depressive symptoms and MDD with acceptable reliability and validity, the level of concordance between these methods is subject to differences among ethnicities (21, 24).

Despite the available evidence on potential differences in the concordance between the CES-D scale and the CIDI-based diagnosis of MDD, very few investigations have explored this subject and none have explored domains of the CES-D scale in-specific as a potential explanation for the differences seen in the CES-D total score. Therefore, we used the National Survey of American Life (NSAL) data to test the moderating effect of ethnicity on the association between CES-D-based depressive symptoms and CIDI-based depression comparing Blacks and Whites. We explored potential ethnic differences in the cross-sectional association between different factors of the CES-D scale and CIDI-based clinical depression.

## Materials and Methods

Data came from the NSAL, 2001–2003. As the most comprehensive study on mental health of African-Americans (28, 29), the NSAL had applied a national household probability sampling of adults (18 years old and older) and adolescents (13–17 years old). The study includes “*detailed measures of health; social conditions; stressors; distress; subjective, observational and objective neighborhood conditions; and social and psychological protective and risk factors*” as well as CIDI-based diagnoses of mental disorders (29). More technical information about the NSAL is documented elsewhere (28–30). In this study, we focused on African-Americans and non-Hispanic Whites.

### Participants

As a nationally representative household survey, the NSAL included 3570 African-Americans, 1623 Caribbean Blacks, 1006 non-Hispanic White adults, and 1200 African-American and Afro-Caribbean adolescents aged 13–17 residing in the 48 conterminous states (29). The NSAL study distinguished African-Americans as Blacks with no identified ancestral tie in the Caribbean. Non-Hispanic Whites included Caucasian adults with no Hispanic ancestry reported (31). African-Americans were sampled from large cities and other urban/rural areas. Non-Hispanic Whites were sampled in proportion to Blacks from the “same contexts and geographical areas” in order to optimize for comparative descriptive and multivariate analyses in the Black/White statistical contrast (29). Included in the sample of non-Hispanic Whites is a subsample of 115 individuals who are subject to large sampling error and are not considered in most analyses (29). Overall, we included 3570 African-Americans and 891 non-Hispanic Whites in our study. A full description of methods, including sampling, has been described elsewhere (28–31).

### Interview

About 86% of the interviews were face to face or computer assisted, and the remaining 14% were telephone interviews. Each interview lasted about 2 h and 20 min. All interviews were conducted in English. The final overall response rate was 70.7% for African-Americans and 69.7% for non-Hispanic Whites.

### Measures

#### Depressive Symptoms

In the NSAL, an abbreviated version of CES-D composed of 12 items was applied with each item scoring from 1 to 3. The items evaluate major aspects of a person’s depressive symptoms, including somatic symptoms (i.e., my sleep was restless), negative affect (i.e., I felt depressed), positive affect (i.e., I was happy), and interpersonal problems (i.e., people were unfriendly) where usually the somatic symptoms and negative affect are considered as a single unified factor (Table A1). More information on validity and reliability of the CES-D is available elsewhere (13–15).

### Outcomes

#### Depressive Diagnoses

Outcomes are lifetime major depressive disorder (MDD), lifetime major depressive episode (MDE), 12-month MDE, 30-day MDE, and 30-day major depressive disorder with hierarchy (MDDH). In the NSAL, the CIDI of the World Mental Health project was applied. The CIDI is a tool for diagnosis of recent and lifetime mental disorders by trained interviewers (2). CIDI has shown a good concordance with the SCID diagnoses (3–5).

### Ethics

The survey received institutional approval by the Review Board of the University of Michigan according to The Code of Ethics of the World Medical Association (Declaration of Helsinki, Edinburgh 2000 revision). All participants provided informed consent to participate in the survey.

### Statistical Analysis

We used Stata version 13 (Stata Corp., College Station, TX, USA) for data analysis. As the NSAL had a complex sampling design (a multistage sample design involving clustering and stratification), we applied appropriate statistical techniques, which account for the complex design. In brief, weights were applied based on strata, clusters, and non-response. Sub-population analyses for surveys were also applied. Chi^2^ and *t*-test were used for bivariate analyses where appropriate.

Different CES-D domains were the independent variables, and lifetime MDD, lifetime MDE, 12-month MDE, 30-day MDE, and 30-day MDDH were the outcomes. First, we fitted ethnicity-specific logistic regressions for data analysis. Then, we ran three models for each CES-D factor or CES-D total score as a predictor in the pooled sample, considering each depressive diagnosis as the outcome; Model 1 only included a CES-D factor or CES-D total score, Model 2 also included ethnicity, and Model 3 also included CES-D factor–ethnicity interaction. Adjusted odds ratios (OR) and 95% confidence intervals (CI) were reported. The *p*-value <0.05 was considered to be statistically significant.

## Results

Table 1 shows the results of descriptive statistics of the CES-D factors and depressive disorders based on ethnicity. According to this table, the CES-D total score and the CES-D factors were higher among Whites than Blacks. In comparison with Blacks, Whites also had a higher prevalence of lifetime MDD [Whites = 19.2 (16.77–21.89), Blacks = 10.31 (9.19–11.54)], lifetime MDE [Whites = 20.19 (17.68–22.94), Blacks = 12.01 (10.81–13.32)], 12-month MDE [Whites = 7.88 (6.49–9.54), Blacks = 6.7 (5.82–7.71)], 30-day MDE [Whites = 3.79 (2.87–4.99), Blacks = 2.41 (1.80–3.22)], and 30-day MDDH [Whites = 3.68 (2.74–4.93), Blacks = 1.9 (1.39–2.57)], while the relevant values for the pooled sample were in between.

**Table 1:** **Descriptive statistics of the CES-D factors and depressive disorders based on ethnicity**.

	All		Blacks		Whites	
	Mean	95% CI	Mean	95% CI	Mean	95% CI
**CES-D domains**						
Total	7.58	7.08–8.09	6.71	6.33–7.09	8.79	7.74–9.84
Positive affect	2.34	2.15–2.53	2.09	1.94–2.25	2.68	2.27–3.10
Negative affect	4.28	3.98–4.57	3.71	3.49–3.92	5.06	4.50–5.63
Interpersonal problems	0.97	0.90–1.03	0.91	0.85–0.98	1.04	0.90–1.18
**Lifetime MDD**						
No	85.07	83.35–86.65	89.69	88.46–90.81	80.80	78.11–83.23
Yes	14.93	13.35–16.65	10.31	9.19–11.54	19.20	16.77–21.89
**Lifetime MDE**						
No	83.75	82.05–85.31	87.99	86.68–89.19	79.81	77.06–82.32
Yes	16.25	14.69–17.95	12.01	10.81–13.32	20.19	17.68–22.94
**12-month MDE**						
No	92.68	91.76–93.52	93.30	92.29–94.18	92.12	90.46–93.51
Yes	7.32	6.48–8.24	6.70	5.82–7.71	7.88	6.49–9.54
**30-day MDE**						
No	96.88	96.20–97.43	97.59	96.78–98.20	96.21	95.01–97.13
Yes	3.12	2.57–3.80	2.41	1.80–3.22	3.79	2.87–4.99
**30-day MDDH**						
No	97.18	96.49–97.73	98.10	97.43–98.61	96.32	95.07–97.26
Yes	2.82	2.27–3.51	1.90	1.39–2.57	3.68	2.74–4.93

*CES-D, Center for Epidemiologic Studies Depression scale; MDD, major depressive disorder; MDE, major depressive episode; MDDH, major depressive disorder with hierarchy*.

Table 2 represents the results of logistic regressions on the associations of the CES-D total score and the CES-D factors with depressive disorders based on ethnicity. The CES-D total score and the CES-D positive affect were positively associated with odds of lifetime MDD, lifetime MDE, 12-month MDE, 30-day MDE, and 30-day MDDH among Blacks and Whites with relatively similar OR values. The CES-D negative affect and the CES-D interpersonal problems were positively associated with lifetime MDD and lifetime MDE in both Blacks and Whites; however, the OR values were considerably higher in Blacks. The association between CES-D scores of negative affect and interpersonal problems with CIDI diagnosis of 12-month MDE, 30-day MDE, and 30-day MDDH were statistically positive for Blacks but not for Whites.

**Table 2:** **Summary of regressions on the associations between CES-D factors and depressive disorders based on ethnicity**.

	Blacks		Whites	
	OR	95% CI	OR	95% CI
**Lifetime MDD**				
CES-D total	1.10***	1.08–1.13	1.10***	1.06–1.15
CES-D positive affect	1.16***	1.11–1.20	1.17**	1.05–1.30
CES-D negative affect	1.17***	1.13–1.22	1.01**	1.01–1.02
CES-D interpersonal problems	1.20***	1.11–1.31	1.04***	1.02–1.07
**Lifetime MDE**				
CES-D total	1.13***	1.10–1.15	1.11***	1.08–1.15
CES-D positive affect	1.19***	1.14–1.25	1.19***	1.09–1.30
CES-D negative affect	1.20***	1.16–1.24	1.01*	1.00–1.02
CES-D interpersonal problems	1.28***	1.16–1.40	1.04**	1.01–1.06
**12-month MDE**				
CES-D total	1.21***	1.18–1.24	1.19***	1.10–1.28
CES-D positive affect	1.33***	1.26–1.41	1.20***	1.10–1.31
CES-D negative affect	1.32***	1.27–1.37	1.01	0.99–1.02
CES-D interpersonal problems	1.46***	1.34–1.60	1.01	0.97–1.06
**30-day MDE**				
CES-D total	1.21***	1.17–1.26	1.21***	1.15–1.27
CES-D positive affect	1.37***	1.25–1.49	1.23***	1.14–1.32
CES-D negative affect	1.33***	1.27–1.40	1.01	0.99–1.03
CES-D interpersonal problems	1.40***	1.28–1.54	1.03	0.97–1.09
**30-day MDDH**				
CES-D total	1.20***	1.15–1.25	1.19***	1.13–1.26
CES-D positive affect	1.34***	1.22–1.47	1.21***	1.14–1.29
CES-D negative affect	1.32***	1.25–1.40	1.01	0.99–1.03
CES-D interpersonal problems	1.40***	1.26–1.56	1.03	0.97–1.09

*CES-D, Center for Epidemiologic Studies Depression scale; MDD, major depressive disorder; MDE, major depressive episode; MDDH, major depressive disorder with hierarchy*.

*p < 0.05.

**p < 0.01.

***p < 0.001.

Tables 3–5 show the summary of stepwise regressions on the associations of the CES-D factors and ethnicity with lifetime depressive diagnoses in the pooled sample. According to the regressions for CES-D total score, the total score showed positive association with all depressive diagnoses in all three models. The association of Black ethnicity with depressive diagnoses was significantly negative only for lifetime MDD and lifetime MDE. No significant interaction was detected in any depressive diagnosis.

**Table 3:** **Summary of regressions on the associations between CES-D factors and ethnicity on lifetime depressive disorders in the pooled sample**.

	Model 1		Model 2		Model 3	
	OR	95% CI	OR	95% CI	OR	95% CI
**Lifetime MDD**						
**Total score**						
CES-D total score	1.11***	1.09–1.13	1.10***	1.08–1.13	1.10***	1.06–1.14
Blacks	–	–	0.68**	0.52–0.88	0.67	0.38–1.19
CES-D total score × Blacks	–	–	–	–	1.00	0.96–1.04
**Positive affect**						
CES-D positive affect	1.17**	1.11–1.24	1.16***	1.10–1.23	1.16***	1.10–1.23
Blacks	–	–	0.62***	0.50–0.78	0.62***	0.50–0.78
CES-D positive affect × Blacks	–	–	–	–		
**Negative affect**						
CES-D negative affect	1.02**	1.02–1.03	1.02***	1.01–1.03	1.01***	1.01–1.02
Blacks	–	–	0.62***	0.49–0.78	0.30***	0.22–0.41
CES-D negative affect × Blacks	–	–	–	–	1.16***	1.11–1.20
**Interpersonal problems**						
CES-D interpersonal problems	1.07***	1.05–1.09	1.05****	1.03–1.07	1.04***	1.02–1.07
Blacks	–	–	0.61***	0.49–0.78	0.51***	0.39–0.67
CES-D interpersonal problems × Blacks	–	–	–	–	1.15***	1.06–1.26
**Lifetime MDE**						
**Total score**						
CES-D total score	1.12***	1.11–1.14	1.12***	1.10–1.14	1.11***	1.08–1.15
Blacks	–	–	0.74*	0.58–0.95	0.65	0.38–1.12
CES-D total score × Blacks	–	–	–	–	1.01	0.98–1.05
**Positive affect**						
CES-D positive affect	1.20***	1.14–1.26	1.19***	1.13–1.25	1.19***	1.13–1.25
Blacks	–	–	0.67***	0.55–0.83	0.67***	0.55–0.83
CES-D positive affect × Blacks	–	–	–	–		
**Negative Affect**						
CES-D negative affect	1.02***	1.02–1.03	1.02***	1.01–1.03	1.01**	1.00–1.02
Blacks	–	–	0.67**	0.52–0.87	0.28***	0.20–0.40
CES-D negative affect × Blacks	–	–	–	–	1.19***	1.15–1.23
**Interpersonal problems**						
CES-D interpersonal problems	1.06***	1.04–1.08	1.05***	1.02–1.07	1.04**	1.01–1.06
Blacks	–	–	0.67**	0.53–0.84	0.51***	0.40–0.66
CES-D interpersonal problems × Blacks	–	–	–	–	1.23***	1.12–1.35

*CES-D, Center for Epidemiologic Studies Depression scale; MDD, major depressive disorder; MDE, major depressive episode*.

*p < 0.05.

**p < 0.01.

***p < 0.001.

**Table 4:** **Summary of regressions on the associations between CES-D factors and ethnicity on 12-month depressive disorders in the pooled sample**.

	Model 1		Model 2		Model 3	
	OR	95% CI	OR	95% CI	OR	95% CI
**12-month MDE**						
**Total score**						
CES-D total score	1.20***	1.16–1.24	1.20***	1.16–1.24	1.19***	1.11–1.27
Blacks	–	–	1.12	0.71–1.76	0.85	0.25–2.94
CES-D total score × Blacks	–	–	–	–	1.02	0.95–1.10
**Positive affect**						
CES-D positive affect	1.27***	1.20–1.33	1.27***	1.20–1.34	1.27***	1.20–1.34
Blacks	–	–	0.95	0.67–1.35	0.95	0.67–1.35
CES-D positive affect × Blacks	–	–	–	–		
**Negative affect**						
CES-D negative affect	1.02**	1.01–1.03	1.02*	1.00–1.03	1.01	0.99–1.02
Blacks	–	–	1.05	0.70–1.58	0.21***	0.13–0.34
CES-D negative affect × Blacks	–	–	–	–	1.31***	1.26–1.36
**Interpersonal problems**						
CES-D interpersonal problems	1.03*	1.00–1.07	1.03	0.99–1.07	1.01	0.97–1.05
Blacks	–	–	0.94	0.66–1.36	0.54**	0.37–0.79
CES-D interpersonal problems × Blacks	–	–	–	–	1.44**	1.31–1.59

*CES-D, Center for Epidemiologic Studies Depression Scale; MDE, major depressive episode*.

*p < 0.05.

**p < 0.01.

***p < 0.001.

**Table 5:** **Summary of regressions on the associations between CES-D factors and ethnicity on 30-day depressive disorders in the pooled sample**.

	Model 1		Model 2		Model 3	
	OR	95% CI	OR	95% CI	OR	95% CI
**30-day MDE**						
**Total score**						
CES-D total score	1.21***	1.18–1.25	1.21***	1.18–1.25	1.21***	1.15–1.27
Blacks	–	–	0.90	0.52–1.54	0.86	0.25–2.95
CES-D total score × Blacks	–	–	–	–	1.00	0.94–1.07
**Positive affect**						
CES-D positive affect	1.29***	1.22–1.37	1.29***	1.21–1.37	1.29***	1.21–1.37
Blacks	–	–	0.80	0.47–1.35	0.80	0.47–1.35
CES-D positive affect × Blacks	–	–	–	–		
**Negative affect**						
CES-D negative affect	1.02***	1.01–1.04	1.02*	1.00–1.04	1.01	1.00–1.03
Blacks	–	–	0.87	0.47–1.64	0.14***	0.07–0.31
CES-D negative affect × Blacks	–	–	–	–	1.32**	1.25–1.39
**Interpersonal problems**						
CES-D interpersonal problems	1.05**	1.01–1.10	1.04	0.99–1.10	1.03	0.98–1.09
Blacks	–	–	0.77	0.44–1.36	0.48*	0.27–0.86
CES-D interpersonal problems × Blacks	–	–	–	–	1.36***	1.22–1.51
**30-day MDDH**						
**Total score**						
CES-D total score	1.20***	1.16–1.24	1.20***	1.16–1.24	1.19***	1.131.26
Blacks	–	–	0.71	0.40–1.25	0.63	0.18–2.18
CES-D total score × Blacks	–	–	–	–	1.01	0.94–1.08
**Positive affect**						
CES-D positive affect	1.27***	1.21–1.34	1.26***	1.19–1.34	1.26***	1.19–1.34
Blacks	–	–	0.64	0.37–1.12	0.64	0.37–1.12
CES-D positive affect × Blacks	–	–	–	–		
**Negative affect**						
CES-D negative affect	1.03***	1.01–1.04	1.02*	1.00–1.04	1.01	1.00–1.03
Blacks	–	–	0.69	0.36–1.31	0.12***	0.05–0.029
CES-D negative affect × Blacks	–	–	–	–	1.30***	1.23–1.39
Interpersonal problems						
CES-D interpersonal problems	1.06**	1.02–1.11	1.04	0.99–1.10	1.03	0.98–1.09
Blacks	–	–	0.62	0.34–1.13	0.39**	0.21–0.73
CES-D interpersonal problems × Blacks	–	–	–	–	1.35**	1.20–1.53

*CES-D, Center for Epidemiologic Studies Depression scale; MDE, major depressive episode; MDDH, major depressive disorder with hierarchy*.

*p < 0.05.

**p < 0.01.

***p < 0.001.

According to the regressions for CES-D positive affect factor, the positive affect showed positive association with all depressive diagnoses in all three models. The association of African-American ethnicity with depressive diagnoses was significantly negative only for lifetime MDD and lifetime MDE. No significant interaction was detected in any depressive diagnosis.

According to the regressions for CES-D negative affect factor, the negative affect showed positive association with lifetime MDD and lifetime MDE in all three models, and with other diagnoses in Model 1 and Model 2. The association of African-American ethnicity with depressive diagnoses was negative for lifetime MDD and lifetime MDE, while for other depressive diagnoses, the association was non-significant in Model 2 and negative in Model 3. A positive interaction was detected in all depressive diagnoses.

According to the regressions for CES-D interpersonal problems factor, the interpersonal problems showed a positive association with lifetime MDD and lifetime MDE in all three models, and with other diagnoses in Model 1. The association of African-American ethnicity with depressive diagnoses was negative for lifetime MDD and lifetime MDE, while for other depressive diagnoses, the association was non-significant in Model 2 and negative in Model 3. A positive interaction was detected in all depressive diagnoses.

## Discussion

We found ethnic differences in the cross-sectional associations between the CES-D negative affect and the CES-D interpersonal problems factors and clinical depression diagnoses, suggesting stronger associations among Blacks compared to Whites. Thus, at each given point of time, negative affect and interpersonal problems were more strongly associated with MDD among Black than Whites, which may be due to higher severity of depressive symptoms among Blacks who endorsement of criteria for clinical depression. Similar differences could not be found for the CES-D total score and the CES-D positive affect, implying that CES-D total score may fail to reveal ethnic differences in the presence of MDD in diverse populations.

There is an ongoing question if CES-D and CIDI similarly reflect depression among Blacks and Whites (32, 33). Clinical conditions and psychosocial issues that are differently distributed among Blacks and Whites (34–38) may affect the reliability and validity of depressive symptoms (12, 17, 39, 40). Some studies have reported lower reliability coefficients for CES-D in Blacks compared with Whites (12, 41). Other studies have reported higher reliability coefficients for the negative affect domain and the interpersonal problems domain of CES-D in Blacks compared with Whites (10, 39, 40). In another study, the reliability of the CES-D scale was higher in Blacks across all domains when compared to Anglos and Hispanics (17). In addition, it seems that although there seems to be similar centrality of items in both non-DSM depressive symptoms, such as CES-D, and DSM-based diagnostic methods, such as the CIDI (42), the CES-D scale may reflect more of a person’s interrelated conditions, including chronic health conditions and socioeconomic factors when compared with the CIDI (43). Some evidence show that factorial structure of the CES-D scale is relatively invariant across races and ethnicities (11, 12, 17, 20, 39–41); however, it has been suggested that certain items of the CES-D are group specific (10, 11, 41, 44). African-Americans have shown higher rates of endorsement of some items, including “unfriendly” compared to Whites (10, 41).

Previous research has shown that the degree of concordance between the perception of mental health and CIDI-based diagnosis of MDD may be influenced by race and ethnicity (22, 23, 45). In other words, how psychiatric disorders are reflected through perception of distress may be altered by race and ethnicity (23). As an example, in comparison with Blacks, Whites have shown a weaker association of perceived mental health and psychiatric disorders, including MDD (22). Interestingly, ethnicity may influence the reflection of DSM-based psychiatric disorders through perceived mental health (23). On the other hand, Blacks have shown weaker associations of perceived mental health distress with the CES-D scores in comparison with Whites (45). In line with this finding, differences have been reported between Blacks and Whites in the perception of interpersonal interactions and consequently the CES-D scale scores (10, 19, 46). In other words, significant measurement bias has been detected in the ­interpersonal domain of the CES-D scale considering ethnicity (47, 48), and the previous research has shown higher reliability for the negative affect domain and interpersonal problems domain of the CES-D in Blacks compared with Whites (12). Further research has detected differences in the frequency of endorsement of single items between Blacks and Whites (46). Therefore, race and ethnicity may change the link between depressive disorders, such as MDD and the CES-D scale score as a measure of depressive symptoms (34).

There are inconsistencies in the previous studies on correlates of depressive symptoms and clinical depression, including self-rated health, chronic medical conditions (CMCs), obesity, mortality, and SRMH in Blacks and Whites (22, 23, 37, 38, 45, 49, 50). In general, Blacks have shown higher rates of CMCs, mortality, and lower self-rated health compared to the Whites, while they have shown lower rates of clinical depression. In addition, there are differences between Blacks and Whites in the association of depression with cardiovascular diseases (50) and the predicting role of depressive symptoms for changes in CMCs over time (37). Furthermore, a study has shown that a poor SRMH score is a stronger predictor of CIDI-based anxiety and mood disorders, including depressive type disorders, in elder Whites rather than Blacks (22). Although it seems that the etiology of ethnic difference in the association between SRMH and CIDI-based diagnosis of mental disorders is unknown, a number of explanations have been suggested. First, in comparison with Whites, elder Blacks may be less likely to recognize their symptoms as representative of underlying mental health issues (22, 45). Second, Whites and Blacks may have different points of reference in evaluating mental health status (22, 45). Third, there may be culture specific symptoms that our current measures do not capture properly (22). Interestingly, in our recently published study, we found that depressive symptoms predicted subsequent CIDI based clinical depression 15 years later in Whites but not Blacks (24). Building upon our previous study by exploring the domains of depressive symptoms in specific, results of the current study may contribute to a better understanding of the mentioned findings concerning SRMH and the CES-D scale as self-rated measures of depressive symptoms with frequent application in population studies. The fact that we found positive interactions for Black ethnicity with negative affect and interpersonal problems domains for predicting depressive disorders highlights the importance of explaining the observed differences in concordance between SRMH and CES-D with CIDI based diagnosis of depression by considering ethnic correlates, including culture and perception, for each domain in specific.

This study has implications both for clinical practice, public health practice and research. Race should be considered while CIDI and CES-D are applied for diagnosis of clinical depression or depressive symptoms among diverse populations. There may be a gap in the level of concordance between self-reported measures of depression, such as the CES-D scale, and the gold standard survey instruments, such as CIDI, which are both widely used in epidemiological studies. Causes of discordance can be mainly attributed to the self-measured scales at least in some domains assessing interpersonal issues rather than the flaws in approved methods of diagnosis, such as CIDI, which should be taken into consideration when relying on self-rated measures in population studies of ethnic groups. Whether this discordance is a matter of difference in characteristics of depression between Blacks and Whites, including cultural correlates, perception, and socioeconomic status (SES), or it is a matter of the design of the measures (CES-D or CIDI) remains to be scrutinized in further research. In addition, consequent to past and present evidence on the discordance of the CES-D scale and the CIDI, the level of certainty up to which the decisions about a diagnosis are made, whether in a clinical setting or in a public health setting, should be carefully evaluated when using the CES-D scale rather than gold standard methods such as the CIDI.

Finally, we found interactions between ethnicity and a number of CES-D domains on endorsement of CIDI-based clinical depression. There are some previous studies on factorial structure of the original 20-item CES-D scale concerning Blacks and Whites (20, 39–41). While the popular 4-factor models have yielded high overall fits for both ethnicities in these studies, there are inconsistent differences in the factor loadings between Blacks and Whites with interpersonal items having higher loadings in Blacks (20, 41). Further research is recommended on this issue.

Our study is subject to a number of limitations. First, we did not control for covariates, such as age, gender, socioeconomic status, and physical health. Second, we did not account for psychiatric disorders that tend to be comorbid with clinical depression. Third, we did not use the original CES-D but a 12-item CES-D scale. However, this is one of the first studies on the ethnic comparison of the association between the CES-D domains and clinical depression based on the CIDI in a large sample. Finally, we used a nationally representative sample, which adds to the strength of our study.

In conclusion, our findings suggest that Blacks and Whites differ in the magnitude of cross-sectional association between some of the CES-D domains (i.e., negative affect and interpersonal problems) and CIDI-based endorsement of clinical depression in the general population. Thus, negative affect and interpersonal problems domains of the CES-D more strongly predict presence of a depressive disorder among Blacks compared to Whites. Ethnic differences in concordance between these two CES-D domains and endorsement of criteria for depressive disorders based on CIDI suggest that there are complex links between ethnicity, depressive symptoms, depression, and psychosocial well-being overall.

## Author Contributions

SA: concept, design, analysis, contribution to the revision, and approval of the final draft. EM-Z: extensive literature review, first draft, contribution to the revision, and approval of the final draft.

## Conflict of Interest Statement

The authors declare that the research was conducted in the absence of any commercial or financial relationships that could be construed as a potential conflict of interest.

## Appendix

**Table A1:** **Factors of the 12-item Center for Epidemiologic Studies Depression (CES-D) Scale**.

	Reversed
**Negative affect/somatic symptoms**	
I had trouble keeping my mind on what I was doing	−
I felt depressed	−
I felt that everything I did was an effort	−
My sleep was restless	−
I had crying spells	−
I could not get “going”	−
**Positive affect**	
I felt hopeful about the future	+
I felt that I was just as good as other people	+
I was happy	+
I enjoyed life	+
**Interpersonal problems**	
People were unfriendly	−
I felt that people disliked me	−

*CES-D, Center for Epidemiologic Studies Depression Scale*.
